# Intermittent Preventive Therapy with Sulfadoxine-Pyrimethamine for Malaria in Pregnancy: A Cross-Sectional Study from Tororo, Uganda

**DOI:** 10.1371/journal.pone.0073073

**Published:** 2013-09-04

**Authors:** Emmanuel Arinaitwe, Veronica Ades, Andrew Walakira, Boaz Ninsiima, Olive Mugagga, Teja S. Patil, Alanna Schwartz, Moses R. Kamya, Sussann Nasr, Michelle Chang, Scott Filler, Grant Dorsey

**Affiliations:** 1 Infectious Disease Research Collaboration, Kampala, Uganda; 2 Department of Obstetrics, Gynecology & Reproductive Sciences, University of California San Francisco, San Francisco, California, United States of America; 3 Department of Medicine, University of California San Francisco, San Francisco, California, United States of America; 4 Department of Medicine, Makerere University College of Health Sciences, Kampala, Uganda; 5 Centers for Disease Control and Prevention, Atlanta, Georgia, United States of America; 6 The Global Fund to Fight Acquired Immune Deficiency Syndrome, Tuberculosis, and Malaria, Geneva, Switzerland; Menzies School of Health Research, Australia

## Abstract

**Background:**

Intermittent preventive treatment during pregnancy (IPTp) with sulfadoxine-pyrimethamine (SP) is widely recommended in sub-Saharan Africa to reduce the risk of malaria and improve birth outcomes. However, there are reports that the efficacy of IPTp with SP is waning, especially in parts of Africa where antimalarial resistance to this drug has become widespread.

**Methodology/Principal Findings:**

We conducted a cross-sectional study of 565 HIV-uninfected women giving birth at Tororo District Hospital in southeastern Uganda. The primary objective of the study was to measure associations between use of SP during pregnancy from antenatal records and the risk of adverse outcomes including placental malaria, low birth weight, maternal parasitemia and maternal anemia. The proportion of women who reported taking 0, 1, 2, and 3 doses of SP during pregnancy was 5.7%, 35.8%, 56.6% and 2.0% respectively. Overall, the prevalence of placental malaria was 17.5%, 28.1%, and 66.2% by placental smear, PCR, and histopathology, respectively. In multivariate analyses controlling for potential confounders, ≥2 doses of SP was associated with non-significant trends towards lower odds of placental malaria by placental smear (OR = 0.75, p = 0.25), placental malaria by PCR (OR = 0.93, p = 0.71), placental malaria by histopathology (OR = 0.75, p = 0.16), low birth weight (OR = 0.63, p = 0.11), maternal parasitemia (OR = 0.88, p = 0.60) and maternal anemia (OR = 0.88, p = 0.48). Using a composite outcome, ≥2doses of SP was associated with a significantly lower odds of placental malaria, low birth weight, maternal parasitemia, or maternal anemia (OR = 0.52, p = 0.01).

**Conclusions/Significance:**

In this area of Uganda with intense malaria transmission, the prevalence of placental malaria by histopathology was high even among women who reported taking at least 2 doses of SP during pregnancy. The reported use of ≥2 doses of SP was not associated with protection against individual birth and maternal outcome measures but did protect against a composite measure of any adverse outcome.

## Introduction

Malaria in pregnancy remains a widespread problem in sub-Saharan Africa, where approximately one in four pregnant women has evidence of infection with *Plasmodium falciparum* at the time of delivery [1]. Placental malaria is associated with several adverse outcomes including intrauterine growth restriction, preterm delivery, low birth weight (LBW), stillbirth, early neonatal death, and maternal anemia [1]. To reduce the burden of malaria and improve birth outcomes, intermittent preventive treatment in pregnancy (IPTp) with the drug sulfadoxine-pyrimethamine (SP) is recommended by the World Health Organization and has been widely adopted as a cornerstone of malaria control in most African countries. A number of randomized controlled trials and observational studies in the 1990s demonstrated the efficacy of IPTp with SP in reducing the burden of malaria and improving birth outcomes [2]–[5]. Older recommendations were that pregnant women take at least 2 doses of SP during pregnancy, but recently the WHO has updated their recommendations that SP be provided at each scheduled focused antenatal-care visit in the second and third trimesters [6].

Despite the widespread adoption of IPTp with SP as policy in Africa, there is concern for the continued efficacy of this intervention due to the spread of resistance, especially in East Africa. Sulfadoxine-pyrimethamine belongs to the antifolate class of antimalarial drugs and resistance is mediated by the ordered accumulation of point mutations in the dihydrofolate reductase (*dhfr*) and dihydropteroate synthase (*dhps*) genes. The *dhfr/dhps* quintuple mutant has been strongly associated with SP treatment failure and has recently reached levels >90% in parts of East Africa [7], [8]. Sulfadoxine-pyrimethamine is no longer recommended for treatment of malaria and recent studies have shown poor efficacy when used for the prevention of malaria in pregnant women and infants in Tanzania [9], [10] and school aged children in Uganda [11].

To assess the current effectiveness of IPTp with SP we conducted a cross-sectional study among women giving birth in an area of southeastern Uganda characterized by high malaria transmission intensity and over 90% prevalence of the *dhfr/dhps* quintuple mutant associated with resistance to SP [7]. SP use was documented from antenatal cards and associated with various measures of placental malaria, maternal anemia, and birth outcomes.

## Methods

### Ethics Statement

The study protocol was approved by the Uganda National Council of Science and Technology and the institutional review boards of the University of California, San Francisco, Makerere University, and the U.S. Centers for Disease Control and Prevention. Written informed consent was sought from all participants before being enrolled in the study.

### Study Site, Participants and Design

This study was conducted in Tororo, an area of southeastern Uganda with high malaria transmission intensity and an entomological inoculation rate recently estimated to be 125 infectious bites per person per year in 2011–12 (Grant Dorsey, personal communication). Study participants were women giving birth at Tororo District Hospital (TDH), a government hospital that provides antenatal services and free HIV testing to all pregnant women. Using a cross-sectional study design, all pregnant women with singleton births delivering at TDH who were known to be HIV-uninfected were screened for enrollment if they delivered between Monday 8∶00 am through Friday 4∶00 pm from February 28^th^ through July 4^th^, 2011. Women were enrolled if they fulfilled the following selection criteria: 1) SP use documented from antenatal card if attended antenatal care, 2) HIV status known and negative, 3) absence of reported antimalarial therapy other than SP in the previous 1 month, and 4) provision of informed consent. A standardized questionnaire was administered to all enrolled women including review of their government issued antenatal card. Information collected as part of the questionnaire included demographics, previous pregnancies, bednet use, education level, ownership of household items, and the number and timing of doses of SP (for which administration is directly observed in the antenatal clinic) and other medications. Delivery outcomes were assessed and birth weight obtained using a digital scale (Seca, Birmingham, U.K.). Data on gestational age was not collected because information on last menstrual period was often missing and if present thought to be inaccurate. Biological samples collected included maternal finger prick for blood smear and hemoglobin measurement and placental blood and tissue biopsy.

### Laboratory Methods

Hemoglobin measurements from maternal blood were made using a portable spectrophotometer (HemoCue, Ängelholm, Sweden). Maternal and placental thick blood smears were stained with 2% Giemsa for 30 minutes and examined for malaria parasites by standard microscopy. Parasite density was estimated by counting the number of asexual parasites per 200 white blood cells and calculating parasites per µL, assuming a white blood cell count of 8,000 cells per µL. A smear was judged to be negative if no parasites were seen after review of 500 high-powered fields. Final microscopy results were based on a rigorous quality control system with re-reading all blood smears by a second microscopist and resolution of any discrepancies by a third microscopist. PCR for the detection of malaria parasites were performed on placental blood stored on filter paper using nested PCR as previously described [12].

Placental biopsies of approximately 1–2 cm×1–2 cm from the maternal side were collected using scissors and placed in 10% neutral buffered formalin. After 24 hours, biopsies were trimmed with a razor blade to 1×1 cm size and formalin replaced with fresh neutral buffered formalin. Following 1–3 months of storage, placental tissue was embedded in paraffin wax, microtome sectioned and stained with 2% Giemsa, and hematoxylin and eosin. Histopathological slides were examined using standard and polarized light microscopy for hemozoin pigment in intervillous fibrin, malaria parasites, and macrophages with hemozoin pigment using standardized criteria as previously described [13].

### Statistical Analysis

Data were double entered in Access (Microsoft Corporation, Redmond, Washington, USA), and analyses performed using STATA (Stata Corp., College Station, Texas, USA). The primary exposure variable of interest was IPTp use with SP as indicated on the participant’s antenatal card. Given the distribution of the number of SP doses reported taken, SP usage was dichotomized into <2 doses vs. ≥2 doses of IPTp. Comparisons of characteristics between the two SP usage groups were made using the chi-squared or t-test. Three definitions of placenta malaria were used: 1) any parasitemia by placental blood smear, 2) detection of parasites by PCR, and 3) any evidence of placental malaria by histopathology. Maternal peripheral parasitemia was defined as a positive blood smear and maternal anemia defined as a hemoglobin level <11 gm/dL. Low birth weight was defined as <2500 gm. Univariate and multivariate analyses of associations between SP usage and outcomes of interest were performed using logistic regression. Covariates adjusted for in multivariate analyses included maternal age, gravidity, bednet use, level of education, transmission season, and a wealth index generated using principal component analysis as previously described [14]. Characteristics used to calculate the wealth index included number of people sleeping in room with participant, and household ownership of electricity, television, mobile phone, radio, bicycle, motorcycle, refrigerator and toilet. A p-value of <0.05 was considered statistically significant.

## Results

### Study Profile and Participant Characteristics

**Figure 1:**
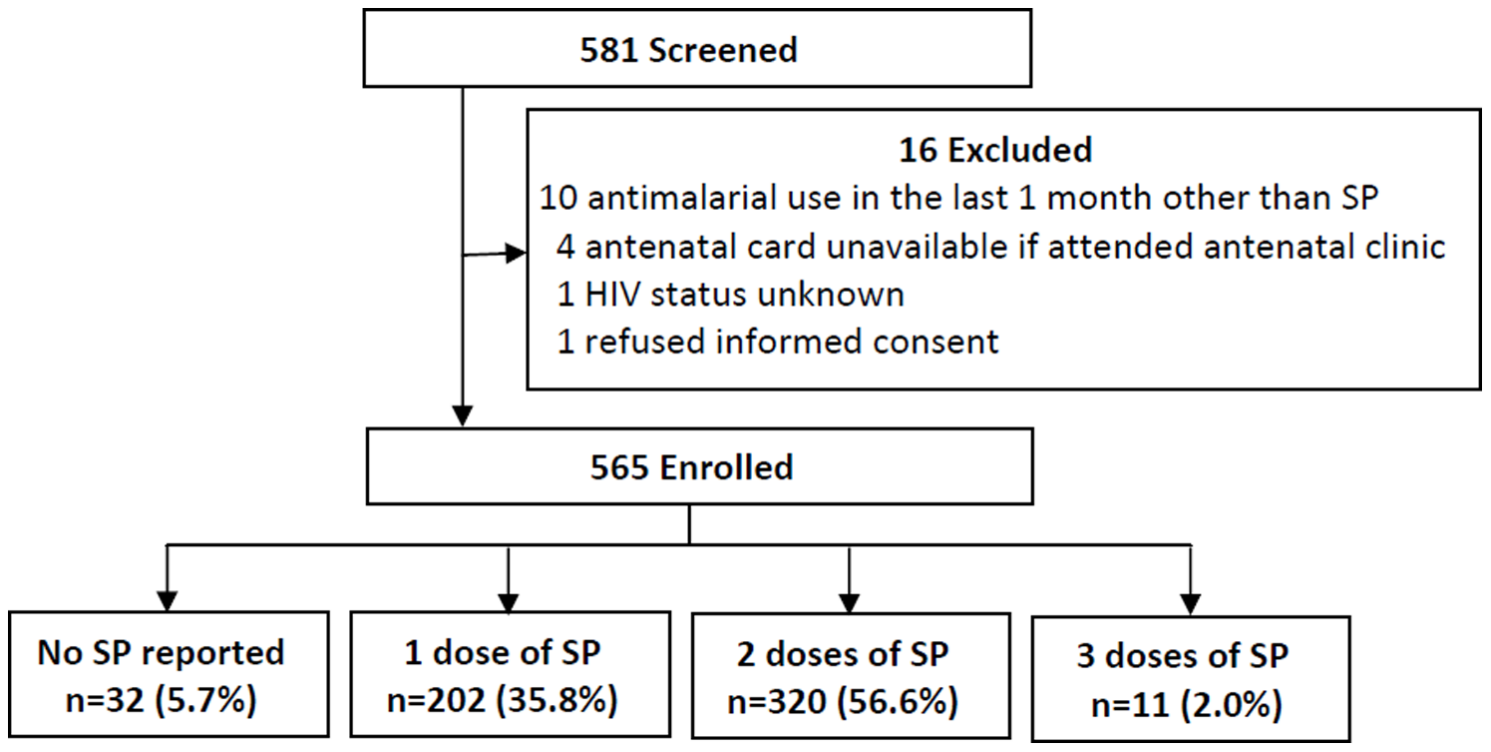
Study profile. SP = sulfadoxine-pyrimethamine.

A total of 581 women were screened and 565 enrolled (Figure 1). Reasons for exclusion included reported antimalarial use in the last month other than SP (n = 10), antenatal card unavailable if attended antenatal clinic (n = 4), HIV status unknown (n = 1), and refusal to provide informed consent (n = 1). Of the 565 women enrolled, 32 (5.7%) women reported to have not taken any SP doses during pregnancy, 202 (35.8%) reported taking 1 dose of SP, 320 (56.6%) reported taking 2 doses of SP, and 11 (2.0%) reported taking 3 doses of SP (Figure 1). Characteristics of study participants stratified by SP usage are presented in Table 1. Maternal age, gravidity, bednet use the prior evening, and delivery during a high transmission season were similar between those with <2 doses of SP and ≥2 doses of SP. Women who reported taking ≥2 doses of SP were more likely to be in the higher wealth index categories (p = 0.008) and had a trend towards a higher level of education (p = 0.06).

**Table 1:** Characteristics of study participants stratified by number of SP doses reported taken.

Characteristic	<2 doses of SP^a^ (n = 234)	≥2 doses of SP^b^ (n = 331)	P-value
Maternal age in years, mean (SD)	24.2 (6.1)	25.0 (6.1)	0.14
Previous pregnancies, n (%)			
0	85 (36.3%)	98 (29.6%)	
1	40 (17.1%)	75 (22.7%)	0.13
≥2	109 (46.6%)	158 (47.7%)	
Reported bednet use the prior evening, n (%)			
None	67 (28.6%)	78 (23.6%)	
Untreated net	16 (6.8%)	19 (5.7%)	0.30
ITN	151 (64.5%)	234 (70.7%)	
Highest level of education completed, n (%)			
None	18 (7.7%)	24 (7.3%)	
Primary school	156 (66.7%)	191 (57.7%)	0.06
Secondary school or greater	60 (25.6%)	116 (51.1%)	
Wealth index, n (%)			
1^st^ quartile	69 (29.5%)	73 (22.1%)	
2^nd^ quartile	69 (29.5%)	79 (23.9%)	0.008
3^rd^ quartile	53 (22.7%)	81 (24.5%)	
4^th^ quartile	43 (18.4%)	98 (29.6%)	
Delivery during high transmission season^c^, n (%)	111 (47.4%)	164 (49.6%)	0.62

a Reported 0 (n = 32) or 1 (n = 202) doses of SP taken during pregnancy.

b Reported 2 (n = 320) or 3 (n = 11) doses of SP taken during pregnancy.

c High transmission season May–June 2011.

### Associations between SP Usage and Outcomes of Interest

Overall the risk of placental malaria as defined by placental blood smear, placental PCR, and histopathology were 17.5%, 28.1%, and 66.2%, respectively. Among 466 placental blood samples that were negative by blood smear, 62 (13.3%) were positive by PCR suggesting the presence of sub-patent parasitemia. Histopathology was the most sensitive measure of placental malaria with 235 of 404 (58.2%) of samples negative for both placental blood smear and PCR having histopathological evidence of placental malaria, the majority (76.2%) of which only had evidence of malarial pigment indicative of past infection. Associations between SP usage and various measures of placental malaria are presented in Table 2. Compared to <2 doses of SP, ≥2 doses of SP was associated with modest trends towards a lower odds of a positive placental blood smear (OR = 0.75, p = 0.25), positive placental PCR (OR = 0.93, p = 0.71), and placental malaria by histopathology (OR = 0.75, p = 0.16), but none of these associations reached statistical significance after controlling for potential confounders. There was no significant interaction between gravidity and associations between SP usage and various measures of placental malaria. Considering only samples with a positive placental blood smear, there was no difference in the geometric mean parasite densities between those with ≥2 doses of SP compared to those with <2 doses of SP (1253 vs. 1267/µL, p = 0.98).

**Table 2:** Associations between use of IPTp-SP and outcomes at delivery.

Outcome	Prevalence of outcomes by IPTp group		Unadjusted OR (95% CI)	P-value	Adjusted^a^ OR(95% CI)	P-value
	<2 doses of SP (n = 234)	≥2 doses of SP (n = 331)				
Positive placental blood smear	49 (20.9%)	50 (15.1%)	0.67 (0.43–1.04)	0.07	0.75 (0.47–1.22)	0.25
Positive placental PCR	71 (30.3%)	88 (26.6%)	0.83 (0.57–1.20)	0.33	0.93 (0.63–1.37)	0.71
Placental malaria by histopathology	168 (71.8%)	206 (62.2%)	0.65 (0.45–0.93)	0.02	0.75 (0.50–1.12)	0.16
Low birth weight (<2500 gm)	30 (12.8%)	26 (7.9%)	0.58 (0.33–1.00)	0.05	0.63 (0.35–1.12)	0.11
Maternal peripheral parasitemia	50 (21.4%)	58 (17.5%)	0.78 (0.51–1.19)	0.25	0.88 (0.56–1.40)	0.60
Maternal anemia (Hb <11 gm/dL)	107 (45.7%)	140 (42.3%)	0.87 (0.62–1.22)	0.42	0.88 (0.62–1.25)	0.48
Composite outcome^b^	208 (88.9%)	263 (79.5%)	0.48 (0.28–0.80)	0.003	0.52 (0.31–0.87)	0.01

a Adjusted for maternal age, gravidity, bednet use, level of education, wealth index, and transmission season.

b Any of the following: placental malaria by any detection method, low birth weight, maternal peripheral parasitemia, or maternal anemia.

**Figure 2:**
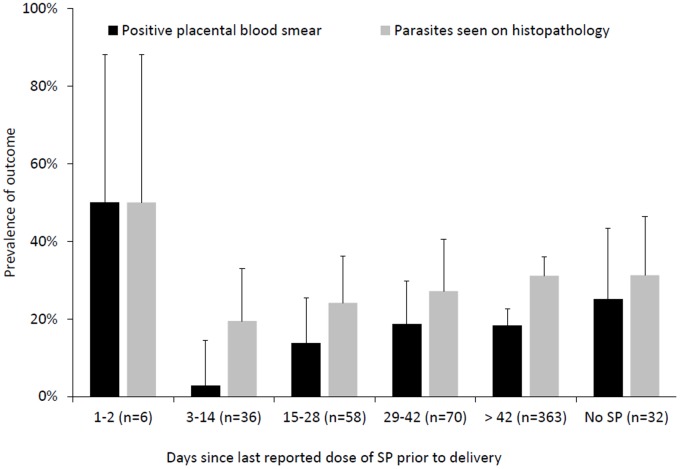
Risk of a positive placental blood smear and active malaria defined as the presence of parasites using histopathology stratified by number of days since last reported dose of SP taken.

Associations between the duration since the last reported dose of SP taken and the risk of a positive placental blood smear and placental malaria by histopathology are presented in Figure 2. Women who reported taking SP 3–14 days prior to delivery had a significantly lower risk of a positive placental blood smear compared to those who reported taking their last dose of SP more than 14 days prior to delivery (2.8% vs. 17.8%, p = 0.02). Similar findings were seen when considering maternal peripheral parasitemia (2.8% vs. 19.6%, p = 0.01). There was no association between the timing of the last reported dose of SP taken and the risk of active malaria defined as the presence of parasites using histopathology (p = 0.48).

Considering birth outcomes, 545 infants survived, 13 were stillborn, and 7 died shortly after birth prior to discharge. Overall the risk of LBW was 9.9%. Compared to <2 doses of SP, ≥2 doses of SP was associated with a modest trend towards a lower odds of LBW (OR = 0.63, p = 0.11), but this did not reach statistical significance after controlling for potential confounders (Table 2). When evaluating birth weight as a continuous outcome, there was no significant difference between mean birth weight for women who reported taking <2 doses of SP vs. ≥2 doses of SP after controlling for potential confounders (3066 vs. 3093 gm, p = 0.12). Considering maternal outcomes, the overall risk of peripheral parasitemia and anemia were 19.1% and 43.7%, respectively. Compared to <2 doses of SP, ≥2 doses of SP was not associated with peripheral parasitemia (OR = 0.88, p = 0.60) or maternal anemia (OR = 0.88, p = 0.48) after controlling for potential confounders. When evaluating maternal hemoglobin as a continuous outcome, there was no significant difference between mean hemoglobin for women who reported taking <2 doses of SP vs. ≥2 doses of SP after controlling for potential confounders (11.1 vs. 11.2 gm/dL, p = 0.94). Based on a composite outcome, ≥2 doses of SP was associated with a significantly lower odds (OR = 0.52, p = 0.01) of any of evidence of placental malaria, LBW, maternal parasitemia, or maternal anemia (Table 2). There was no significant interaction between gravidity and associations between SP usage and various birth outcomes.

## Discussion

In this cross-sectional study of HIV-uninfected women giving birth at a district hospital in Uganda, 94.3% of women had documentation of receiving at least one dose of SP during pregnancy and 58.6% received at least 2 doses of SP. At the time of this study, national recommendations were that women take at least 2 doses of SP during pregnancy. Despite the high coverage of IPTp with SP in this population, the risk of placental malaria was 17.5% by placental blood smear and 66.2% by histopathology. Given the small numbers of women who did not report taking SP or who reported taking >2 doses during pregnancy, comparisons were only made between those with <2 doses of SP versus those with ≥2 doses of SP. Receiving ≥2 doses of SP was not significantly associated with protection against placental malaria, LBW, maternal parasitemia, or maternal anemia, however, there was significant protection when using a composite including any of these adverse outcomes.

IPTp with SP replaced weekly chloroquine prophylaxis for the prevention of malaria in pregnancy in the mid 1990’s and is currently recommended as policy for most countries in sub-Saharan Africa. A systematic review published in 2007 of 4 trials comparing IPTp with 2-dose SP to case management or placebo reported that IPTp with SP was associated with a reduced risk of placental malaria, LBW, and maternal anemia [15]. However, these studies were conducted in areas of low-moderate SP resistance defined as 14-day treatment failure rates of 19–26%. More recent studies have suggested that IPTp with SP may no longer be effective in some areas. In a cross-sectional study conducted from 2002–05 in Tanzania, IPTp with any SP was not associated with protection against placental malaria, LBW, or maternal anemia compared to women who did report taking SP [10]. Of note, this study was conducted in an area where 14-day SP treatment failure rates in children increased from 41% to 68% during the same period [16], [17]. In a large cross-sectional study conducted over a 9 year period in Malawi, IPTp with SP was associated with a decreased risk of placental parasitemia, LBW, and maternal anemia from 1997–2001, but not from 2002–2006 [18]. In two clinical trials from Uganda and Mozambique there were no differences in maternal and fetal outcomes between pregnant women randomized to IPTp with 2-dose SP plus insecticide treated bednets (ITNs) versus ITNs alone [19], [20].

Monitoring the efficacy of IPTp with SP is challenging as it is generally not considered ethical to perform randomized clinical trials with a placebo arm in high transmission areas. Surrogate data from *in vivo* studies of SP for the treatment of symptomatic malaria have been used in the past, however, SP monotherapy is no longer recommended for the treatment of malaria in Africa. The WHO now recommends evaluation of the *in vivo* efficacy of SP in populations with asymptomatic parasitemia as a method of monitoring the effectiveness of IPTp with SP [21]. In a recent study of children with asymptomatic parasitemia from the same area of Uganda where this study was conducted, SP was associated with a decreased risk of parasitemia after 14 days compared to placebo, however, the risk of treatment failure due to recrudescence of resistance parasites was similar when follow-up was extended to 42 days [11]. These results were similar to the findings of this study, where SP appears to have a short-term but temporary benefit in reducing the prevalence of parasitemia detected by microscopy. An alternative method proposed for monitoring the effectiveness of SP is the surveillance of molecular markers of drug resistance. Single-point mutations accumulate in an ordered fashion in the *dhfr* and *dhps* target genes and the prevalence of the *dhfr*/*dhps* quintuple mutant has been correlated with in vivo SP resistance [22]. In pregnant women from Kenya the prevalence of the *dhfr*/*dhps* quintuple mutant increased from 7% in 1996–2000 to 88% in 2008–09 [23]. In studies from Tanzania and our site in Uganda the prevalence of the *dhfr*/*dhps* quintuple mutant has surpassed 90% [7], [24]. In addition, a sixth mutation at *dhps* codon 581 associated with high level SP resistance has now been reported in areas of Tanzania and Kenya [23], [24] but has not been found at our study site in Uganda [7]. Indeed, SP was classified as unsuitable for IPT in eight countries in East Africa based on a geo-referenced database of SP resistance markers [25]. The results of this study and others suggest the efficacy of IPT with SP may be diminishing in East Africa due to the spread of drug resistance.

This study has several limitations. First, we used a cross-sectional study design rather than a placebo-controlled randomized trial since SP for IPTp is the standard of care and withholding it was not felt to be an option. Although we attempted to control for potential confounding factors, the possibility of bias due to unmeasured factors cannot be ruled out, limiting our ability to make causal inferences. Second, SP use was based on documentation from antenatal cards and not measured using a prospective study design. However, reports from antenatal cards are likely accurate given that SP administration is directly observed in the antenatal clinic. Third, over 92% of women in this study reported taking 1–2 doses of SP during pregnancy, therefore we lacked the statistical power to compare outcomes between groups that reported taking no SP or more than 2 doses of SP. Fourth, we were unable to obtain accurate data on gestational age or molecular markers of SP resistance markers, limiting the scope of our analyses. Finally, we cannot rule out the possibility of a type II error, i.e. the possibility that SP provided protection against individual outcomes we were unable to detect due to a lack of statistical power.

In summary, growing evidence from this study and others raise questions about the continued efficacy of IPTp with 2-dose SP for the prevention of placental malaria in areas of East Africa with a high prevalence of SP resistance. Although these data do not provide evidence that IPTp with SP should be abandoned, there is certainly a need to evaluate strategies for chemoprevention in pregnancy that will reduce this risk of placental malaria. Several studies have investigated whether increasing the number of recommended doses of SP would improve efficacy. In a randomized controlled trial from an area with low SP resistance in Mali, 3-dose SP was associated with a lower risk of placental malaria, LBW and preterm birth compared with 2-dose SP [26]. Studies from Malawi in Zambia reported that among HIV-infected and -uninfected women, IPTp with monthly SP was superior to 2-dose SP [27]–[29]. In a recent systematic review and meta-analysis among pregnant women in sub-Saharan Africa, IPTp with 3 or more doses of SP was associated with a higher birth weight and lower risk of LBW than the standard 2-dose regimens [30]. Indeed, recent guidelines from the World Health Organization now recommends IPTp with SP for all pregnant women at each scheduled antenatal visit given at least 1 month apart [6], a policy that has now been adopted in Uganda. Future controlled trials should also evaluate alternatives to SP including mefloquine, artemisinin-based combination therapies, and azithromycin containing combination therapies.
